# An Investigation of the Implicit Endorsement of the Sexual Double Standard Among U.S. Young Adults

**DOI:** 10.3389/fpsyg.2020.01454

**Published:** 2020-06-30

**Authors:** Ashley E. Thompson, Carissa A. Harvey, Katherine R. Haus, Aaron Karst

**Affiliations:** ^1^Department of Psychology, University of Minnesota Duluth, Duluth, MN, United States; ^2^Department of Kinesiology and Health Promotion, University of Kentucky, Lexington, KY, United States; ^3^Department of Psychology, University of Wisconsin–Oshkosh, Oshkosh, WI, United States

**Keywords:** sexual double standard, implicit associations, implicit association test, sexual script theory, sexuality, gender differences

## Abstract

Despite empirical support for the sexual double standard (SDS, in which women are judged more harshly than men for engaging in sexual behavior), recent research has demonstrated inconsistencies, perhaps stemming from biased responding in the self-report measures that are commonly used. As a result, researchers are encouraged to adopt innovative methodological procedures to assess the SDS. Thus, the current study investigated the implicit endorsement of the SDS among 147 young adults (71 men, 76 women) using the Implicit Association Test (IAT) and a limited-awareness gender priming procedure. According to our results, young adults endorsed an implicit preference for sexual stimuli (as compared to neutral stimuli) after receiving a male prime in comparison to a female prime, evidence of an implicit SDS. These results indicate that traditional gendered expectations still exist, in which women are judged more harshly than men for engaging in comparable sexual behavior. Implications and recommendations are outlined for clinicians and researchers working to promote gender equality related to one’s sexuality.

## Introduction

The phenomenon relating to the tendency to hold different standards of sexual conduct for heterosexual women in comparison to heterosexual men is referred to as the sexual double standard (SDS; Marks and Fraley, 2006). In particular, men are less restricted in their sexual freedom and are evaluated more positively as compared to women for engaging in or initiating sexual behavior. In addition, research indicates that men endorse the SDS to a greater extent than women (England and Bearak, 2014), in which 46% of men and 8% of women endorse an SDS when evaluating receptivity to casual sexual offers (Kettrey, 2016).

Scholars have adopted many explanations for the SDS, including (but not limited to): social role theory/sexual script theory (Gaunt, 2012; Farvid et al., 2017), sexual strategies theory (Milhausen and Herold, 2001), and cognitive social learning theory (Mischel, 1966), with each of these theories helping to explain different aspects of the SDS. For example, because sexual strategies theory posits that the SDS stems from discrepant evolutionary pressures facing men and women, it has been used to explain the origins of the SDS. Social role theorists and sexual script theorists posit that the SDS is a result of differing societal expectations of men and women. Thus, social role theory and sexual script theory explain the SDS by including the impact of one’s culture, generation, upbringing, etc. Finally, proponents of cognitive social learning theory argue that the SDS is a byproduct of variations in the reinforcement and punishment men and women receive based on engaging in sexual behaviors that are either consistent or inconsistent with gender roles. Consequently, cognitive social learning theory is used to explain how the SDS is perpetuated (for a review of all theoretical perspectives, see Zaikman and Marks, 2017). Nevertheless, recent studies have identified inconsistencies regarding the continued existence of the SDS, suggesting that it has narrowed or even disappeared entirely (Milhausen and Herold, 2001; Marks and Fraley, 2005). Although this narrowing of the SDS may be a result of equitable sexual socialization of men and women in Western cultures (Petersen and Hyde, 2010), it may also be a byproduct of the methods used to assess SDS endorsement.

In fact, a recent meta-analysis was conducted to investigate whether SDS endorsement was impacted by methodological choices (Endendijk et al., 2019). According to the results, the SDS is still prevalent in today’s society, but varies according to the methodology being used. The authors suggest that endorsement of the SDS might only be present at an implicit level. Thus, researchers are encouraged to refrain from relying exclusively on self-report measures in an effort to reduce outcomes associated with social desirability biases (defined as people’s tendency to “over or understate responses to be represented in a more favorable way”; Haberecht et al., 2015, p. 405) and encouraged to use implicit measures (i.e., those assessing automatic associations between stimuli).

The Implicit Association Test (IAT; Greenwald et al., 1998) is a computer-based classification task in which participants categorize stimuli as quickly as possible according to two target and two attribute concepts, with faster parings indicating greater associative strength. The assumption is that people will respond faster when pairing concepts that are strongly associated than when pairing concepts that are weakly associated.

The IAT is the most widely used and well-validated measure of implicit attitudes (Greenwald et al., 2009) and possesses excellent predictive utility (Richetin et al., 2010). Consequently, because of the need to adopt alternative measures when assessing this phenomenon, the current study used the IAT to assess young adults’ implicit endorsement of the SDS.

## Implicit Endorsement of the Sexual Double Standard

Two studies have adopted the IAT to investigate the implicit endorsement of the SDS (Sakaluk and Milhausen, 2012; Londo, 2017). First, in a study conducted by Sakaluk and Milhausen (2012), 15 young men and 88 young women were instructed to take an IAT that used “male” and “female” as target categories and “sexually positive” and “sexually negative” as attribute categories. Contrary to their hypotheses, men did not endorse an implicit SDS, whereas women endorsed a reverse SDS (in which “female” words were most easily and accurately paired with “sexually positive” words).

Although this study was an important step in using an alternative methodology to assess the SDS, there were a few crucial limitations. First, because only 15 of the 103 participants were men, statistical power was lacking. Second, the sensory modality of the stimuli may have failed to portray their target concept categories of “male” and “female,” which proponents of the IAT argue that visual stimuli (or images) elicit a stronger effect (Greenwald et al., 1998). Finally, because the SDS is an evaluation of two different target concepts (gender and sexuality), the methods used by Sakaluk and Milhausen (2012) may have failed to adequately assess both concepts. The IAT can only accommodate evaluations for one target concept at a time (i.e., gender or sex). To overcome this concern, the two concept categories were collapsed by having participants categorize gendered words to sexually positive and sexually negative words, potentially compromising the construct validity as many of the words selected were not sexual. Examples of the words they used for the “sexually positive” category included “virtuous” and “intelligent.” Words used in their IAT for “sexually negative” included “unacceptable” and “stupid.” Based on their stimuli, their results may reflect broad implicit attitudes toward gender instead of the SDS.

To rectify issues associated with simultaneously assessing associations with two target concepts, a more recent study was conducted in which a priming procedure was administered prior to the IAT (Londo, 2017). In this study, a between-subjects design was adopted in which participants were explicitly primed before the IAT using a paper-based scrambled sentence task. Although there was not a significant effect of the gender of the prime, there were trends suggesting that participants receiving the male prime were more efficiently able to pair sexual stimuli with positive attributes than were participants receiving the female prime. The author argued that the lack of an implicit SDS may relate to the effectiveness of the explicit priming procedure, particularly because implicit associations are least affected by explicit processing (Rydell and McConnell, 2006). The use of an explicit prime to manipulate implicit processing may have failed. It is possible that young adults do implicitly endorse the SDS but that it was not elicited via the explicit priming procedure.

## The Current Study

The current study advanced the literature by assessing young men’s and women’s (ages 18–24) implicit and explicit endorsement of the SDS. Young adults were chosen because sexual scripts are argued to be especially salient during young adulthood (Arnett, 2000). Additionally, as the current study sought to extend and replicate previous research (Sakaluk and Milhausen, 2012; Londo, 2017), a similar target population was recruited.

To address limitations associated with previous research (Sakaluk and Milhausen, 2012; Londo, 2017), the current study recruited a more gender-balanced sample and selected images to represent the target stimuli rather than words^1^. Additionally, the current study incorporated an implicit priming procedure to alleviate potential issues with adopting an explicit priming procedure. An IAT measuring implicit attitudes toward sexuality (categorizing “sexual”/“neutral” stimuli and “positive”/”negative” attributes) was combined with a limited-awareness gender priming task. By incorporating an implicit priming procedure with the use of the IAT, we expected that differences in IAT performance across the two prime conditions would serve as a measure of the SDS (an implicit SDS would be demonstrated if participants showed a stronger implicit preference for sexual stimuli after receiving a male prime as compared to a female prime). It was expected that, although participants would not endorse an explicit SDS (H1), evidence of an implicit SDS would emerge (H2). In addition, men were expected to endorse the SDS to a greater extent than women (H3).

## Materials and Methods

### Participants

The sample size was determined *a priori* using commonly agreed upon cell sizes to achieve adequate statistical power. A total of 165 undergraduate psychology majors were recruited from a mid-sized Midwestern university. Two participants were removed because of their missing response on the gender item and another was omitted because they were older than 24 years of age. This resulted in a sample of 162 young adults (80 men, 82 women), with an average age of 19.21 years (SD = 1.39). The majority identified as heterosexual (94%), White/Caucasian (80%), as currently single (49%), and as having an average of 3.51 sexual partners (SD = 4.32).

### Procedure

Upon arrival to the laboratory, one of four research assistants explained and facilitated the administration of the IAT (incorporating the within-subject limited-awareness priming procedure) and then advised participants to complete the explicit measure, followed by the demographics questionnaire. Participants were then asked one guided debriefing question to determine whether they consciously detected any of the prime images or their features, after which they were debriefed and compensated with course credit. In total, the study took approximately 15 min to complete (with 5 min dedicated to the IAT).

### Mask and Prime Stimuli

The mask stimuli, consisting of a random noise pattern, were generated using MATLAB. To produce the prime stimuli, 20 male and 20 female faces depicting a neutral facial expression were obtained from the Chicago Face Database (Ma et al., 2015). Individual faces were selected to ensure that there were no differences between the average ratings of the male and female faces in terms of perceptions of how *Afraid*, *Angry*, *Happy*, *Sad*, *Surprised*, and *Attractive* they were perceived to be (*ps* = ns). Based on these norms, female faces were rated as being more *feminine* than male faces (*p* < 0.001) and male faces were rated as being more *masculine* than female faces (*p* < 0.001). All faces (hair, face, and ears) were extracted from the original stimuli, converted to grayscale and then placed on the center of a mask stimulus using Adobe^®^ Photoshop CC^®^.

### The Implicit Association Test (Greenwald et al., 1998)

The IAT paradigm was programmed using E Prime 2.0 and was completed via a desktop computer with an 18” CRT monitor. Categorizations were made using a serial button box where the left and rightmost buttons corresponded to target and/or attribute category labels that appeared in the upper left and right-hand side of the screen. Each stimulus remained on the center of the screen until correctly assigned it to a given category.

The IAT comprised of five blocks (blocks three and five collecting the critical data). A masked repetition priming procedure was incorporated into the paradigm on an individual trial basis in blocks three and five, while blocks one, two, and four included the presentation of masks prior to stimulus presentation. Before presenting the stimuli in blocks one, two, and four, three masks were sequentially presented on each trial (150 ms for the first, 17 ms for the second, and 150 ms for the third). Stimulus presentation for blocks three and five proceeded in the same manner, except the second of the three masks was replaced with the prime image for 17 ms. This resulted in a forward and backward masked priming procedure in which the prime image was presented briefly to limit conscious awareness.

The first block required participants to practice sorting visual stimuli depicting the *target* categories: “sexual” (e.g., an image of penetrative sexual behavior) and “neutral” stimuli (e.g., an image of a couple on a bicycle ride). The second block required participants to practice sorting words associated with the *attribute* categories: “positive” and “negative” words. Twenty sorting trials were included in the first and second block. In the third block, participants were asked to sort stimuli from both the *target* and *attribute* categories, with each target and attribute category sharing a side of the screen and a response key. For example, a participant may have been asked to pair “sexual” stimuli with “positive” words and “neutral” stimuli with “negative” words. The fourth block consisted of 30 trials and was identical to the first block; however, the target category labels were switched to appear on opposite sides of the screen. The fifth block was identical to the third block, except participants were now required to associate stimuli with an opposite combination that was employed in block three (e.g., “neutral” and “positive”; “sexual” and “negative”). The third and fifth blocks occurred in counterbalanced order across participants. Both blocks three and five consisted of 120 trials, of which 40 included the male prime, another 40 the female prime (all priming stimuli were presented twice within each block), and 40 trials with a mask instead of the prime (i.e., no-prime trial).

#### Computation of IAT Scores

The IAT generated a difference score (i.e., *D* score) that is calculated using a standard scoring procedure and can be described as an indicator of associative strength (Schnabel et al., 2008). This *D* score is computed using a latency-based algorithm that accounts for response time and trials in which classification errors were made by calculating the difference between the response latencies divided by the total standard deviation (Greenwald et al., 2003). For the current study, latencies that exceeded 10,000 ms were deleted (an indication that participants were distracted). Two different *D* scores were calculated for each participant, one for the female and one for the male prime trials.

After the latency adjustments were made, the error rates were computed and used during the data cleaning process. Because a high error rate may be indicative of a lack of attention or effort, ten participants were excluded because of error rates exceeding 25%. Further, any participant who reported detecting any aspect of the prime during the guided debriefing was excluded from analysis, which resulted in the removal of six additional participants (resulting in a final *N* of 147 young adults).

### Sexual Double Standard Scale (SDSS; Muehlenhard and Quackenbush, 1998; α = 0.70)

The SDSS is a 26-item scale that measures SDS endorsement (e.g., “I question the character of a woman who has a lot of sexual partners”). Participants respond using a four-point Likert scale ranging from 0 (disagree strongly) to 3 (agree strongly). Total scores on the SDSS range from 48 to −30, with 0 indicating no endorsement of an SDS. This scale was scored using a computation method described in detail by Muehlenhard and Quackenbush (1998).

### Demographics Questionnaire

Items assessed gender, age, ethnicity/race, sexual orientation, relationship status, and sexual history.

## Results

First, missing data were evaluated and no participant was missing more than 0.8% of their data. All missing values were treated using listwise deletion. No participants were identified as outliers nor was there significant skew on any variables. According to a sensitivity analysis, the primary analysis (a 2 χ−2 mixed-design factorial ANOVA) with 147 participants was adequately powered (80%) to detect a small-to-medium effect (η*p*^2^ = 0.04) with an alpha = 0.05. These calculations were based on a correlation between the repeated measures of *r* = 0.56. To access the data file, please visit our OSF page given in footnote 1.

### Explicit Endorsement of the Sexual Double Standard

Contrary to our hypothesis (H1), participants endorsed a small explicit SDS as evidenced by a mean SDSS score of 6.65 (SD = 4.35). According to a one-sample *t*-test, participants’ SDSS scores were significantly different from zero, *t*(141) = 18.21, *p* < 0.001, *d* = 1.53, evidencing that young adults still endorse an explicit SDS.

An independent-samples *t*-test was conducted to examine gender differences in the explicit endorsement of the SDS, in which men (*M* = 7.56, SD = 4.84) scored significantly higher on the SDSS than women (*M* = 5.76, SD = 3.64), *t*(128.73) = 2.50, *p* = 0.01, *d* = 0.42. According to these results, both men and women explicitly endorsed the SDS, but men explicitly endorsed it to a greater extent than women.

### Implicit Endorsement of a Sexual Double Standard

To examine implicit endorsement of the SDS, the two *D* scores (*D*_male_, *D*_female_) were used. The mean *D*_male_ score was 0.19 (SD = 0.48) and the *D*_female_ score was 0.13 (SD = 0.50). These scores indicate that the implicit preference for sexual images was stronger when primed with male faces as compared to female faces. According to *D* score effect sizes (slight preference: ±0.15, moderate preference: ±0.35, and strong preference: ±0.65; Greenwald et al., 2003), when receiving the male prime, 57.5% of participants were classified as demonstrating a slight, moderate, or strong preference for sexual images. However, this was reduced to 52.7% when receiving the female prime (see Table 1). To examine implicit endorsement of the SDS as well as gender differences, a 2 (participant gender) χ 2 (type of prime) mixed-design factorial ANOVA was conducted. Consistent with our hypothesis (H2), there was a significant effect of type of prime on IAT performance, *F*(1,144) = 6.48, *p* = 0.01, η*_p_^2^* = 0.04. Participants demonstrated a stronger implicit preference for sexual images in comparison to neutral images when primed with male faces as compared to female faces. Contrary to predictions (H3), the main effect of participant gender (*F*[1,144] = 0.00, *p* = 0.96, η*_p_*^2^ = 0.00) and the interaction effect (*F*[1,144] = 0.21, *p* = 0.65, η*_p_*^2^ = 0.00) were non-significant. Overall, participants endorsed a significant implicit SDS when using the limited awareness gender priming IAT, but the extent to which it was endorsed was unrelated to one’s gender.

**TABLE 1:** Descriptive information for the magnitude of men’s and women’s *D* scores.

	**Men**		**Women**	
	**Male prime *N* (%)**	**Female prime *N* (%)**	**Male prime *N* (%)**	**Female prime *N* (%)**
Strong preference for neutral	7 (9.9%)	6 (8.5%)	3 (4.1%)	6 (8.0%)
Moderate preference for neutral	4 (5.6%)	6 (8.5%)	5 (6.7%)	2 (2.7%)
Slight preference for neutral	7 (9.9%)	6 (8.5%)	8 (10.8%)	10 (13.3%)
No preference	14 (19.7%)	17 (23.9%)	13 (17.6%)	9 (12.0%)
Slight preference for sexual	12 (16.9%)	12 (16.9%)	16 (21.6%)	13 (17.6%)
Moderate preference for sexual	15 (21.1%)	14 (19.7%)	17 (23.0%)	16 (21.6%)
Strong preference for sexual	12 (16.9%)	10 (14.1%)	13 (17.6%)	13 (17.6%)
Total	71 (100.0%)		75 (100.0%)	

*Number and percentage reflect the proportion of young men and women classified into each *D* score. Values may not add up to 100% due to rounding.*

## Discussion

This study was conducted to address discrepancies in the literature investigating the SDS by employing the IAT. Limitations associated with previous research were addressed by incorporating a limited awareness priming procedure. Overall, young adults endorsed both an explicit and an implicit SDS, but gender differences were only present in implicit endorsement.

### Endorsement of an Explicit Sexual Double Standard

Despite research indicating that explicit endorsement of the SDS is disappearing, young adults in the current study endorsed a small explicit SDS which may be related to their geographic location of the sample. Because the sample was comprised of students residing small to mid-sized Midwestern cities, our results may not generalize to other more urban regions, as research reveals that individuals residing in urban regions report more conservative views toward gender and sexuality than those residing in rural/subruban regions (Herek, 2002). Therefore, the SDS likely exists in regions marked by traditional social values and beliefs but not in more progressive areas.

In recognizing the differing views, it is also important to acknowledge the recent charged political climate. The current study was conducted after the most recent presidential election, which spurred a general shift toward a more conservative standpoint, resulting in a reemergence of hostility toward women occupying non-traditional roles (Bock et al., 2017). Thus, it is also possible that we are witnessing resurgence in SDS endorsement according to this mentality.

### Endorsement of an Implicit Sexual Double Standard

In response to the call made by Endendijk et al. (2019), the current study successfully used the IAT and a limited awareness priming procedure to assess the implicit endorsement of the SDS. Our results indicate that young adults implicitly endorse a SDS, in which sexual stimuli were paired more quickly with positive attributes after receiving a male prime in comparison to a female prime. Because existing research reveals that indirectly assessed attitudes influence psychological processes outside of conscious awareness (i.e., spontaneous behavior; Gawronski et al., 2006), the presence of an implicit SDS in the current study may have implications on a person’s behavior and gendered interactions. For example, McConnell and Leibold (2001) found that undergraduate students who implicitly preferred white faces to black faces engaged in more negative spontaneous social interactions with a black experimenter (e.g., leaning away from the experimenter, greater distance between chairs). Thus, based on our data, young adults endorsing an implicit SDS (as compared to those not endorsing an implicit SDS) may spontaneously behave in more negative ways toward women engaging in sexual behavior as compared to men.

The implicit SDS in the current study provides support for the application of sexual strategies theory when explaining the origins of the SDS. For example, proponents of sexual strategies theory posit that men and women experienced different adaptive problems in the sexual domain in our evolutionary past (Buss, 2003). For example, historically men benefited more than women from frequent sex (Buss and Schmitt, 1993), whereas women evolved to be more selective than men when mating (because of greater time investing in offspring). Individuals who behave in ways that counter these reproductive adaptations are likely to be evaluated negatively (Milhausen and Herold, 2001). Thus, despite efforts at the societal level to promote gender equality, the continued endorsement of an implicit SDS is likely instinctual with evolutionary implications, thereby making it resistant to change.

### Gender Differences in the Endorsement of the Sexual Double Standard

Consistent with previous research, gender differences emerged when employing an explicit measure (men endorsed a SDS to a greater extent than did women) but not for the implicit measure. It is possible that this discrepancy can be explained by gender differences associated with the social desirability bias, in which women are more likely to engage in socially desirable responding than are men (Bernardi and Guptill, 2008). Gender differences found using the explicit measure may be more reflective of the increased tendency for women to engage in socially desirable responding than men. Conversely, because the IAT is resistant to self-presentation artifacts, this could explain why no gender differences emerged using the implicit measure.

It is also possible that the gender differences characterizing the explicit data can be explained via social identity theory (Tajfel, 1982) and double standards theory (Foschi, 2000), which posit that people use different standards for making inferences about others based on their social group. In particular, people tend to attribute more positive qualities to members of one’s in-group in comparison to one’s out-group (Abrams and Hogg, 2010). Thus, it is possible that individuals evaluate the sexual behavior of members of their own gender more positively than members of the other gender when completing explicit assessments but not when completing implicit assessments (resulting in a greater explicit SDS for men as compared to women).

### Limitations and Future Directions

Although the current study has advanced our understanding of the SDS, there are a number of limitations worth noting. First, additional forms of validity need to be assessed to establish the generalizability of our results. For example, the extent to which these results predict behavior remains unknown. Second, the participants were young adults attending a Midwestern university. Consequently, the results may fail to generalize and researchers should work to replicate this study with more representative samples of U.S. young adults. Finally, although our results were significant, the effect sizes were fairly small. This may have resulted from a weakened manipulation that was used in an attempt to ensure participants were not consciously aware of the primes (i.e., a presentation time of 17 ms). Future research should modify the paradigm to present multiple primes below awareness within a single trial to strengthen the priming manipulation.

## Conclusion and Implications

The results from the current study contribute to the growing literature on SDS endorsement and have important implications for researchers and educators. For researchers, the incorporation of a priming procedure into the IAT constitutes a novel paradigm that has a plethora of implications. For example, one could determine how preferences for different races, sexualities, genders, etc., are affected by holding different types of information in an active state in the absence of conscious awareness. We hope that others will use this paradigm to advance our scientific understanding of psychological phenomena.

In addition, educators may use the current study results to address gender inequality in our education system, healthcare system, and in our government to eliminate outdated and detrimental perspectives. Additional work could also be done on college campuses to emphasize the normative sexual function of both men and women. By promoting discussion from a progressive and sex-positive standpoint, the equitable treatment of men and women can occur.

## Data Availability Statement

The original contributions presented in the study are publicly available. This data can be found here: https://osf.io/852p6/?view_only=e0d484363f804745b75c9e799adefde8).

## Ethics Statement

The studies involving human participants were reviewed and approved by the REB at the University of Wisconsin Oshkosh. The patients/participants provided their written informed consent to participate in this study.

## Author Contributions

AT was responsible for conceptualizing the study, assisting with the development of all measures, stimuli, and procedures, conducting all data cleaning and analyses, and wrote the majority of the manuscript. CH assisted with writing the manuscript. KH assisted with writing the manuscript. AK oversaw the majority of the development of all measures, stimuli, and procedures, supervised all participant recruitment and data collection, and assisted with the writing of the manuscript. All authors contributed to the article and approved the submitted version.

## Conflict of Interest

The authors declare that the research was conducted in the absence of any commercial or financial relationships that could be construed as a potential conflict of interest.

## Footnotes

1 Supplemental analysis was conducted to examine the relationship between the implicit and explicit measures as well as variations in SDS endorsement according to demographic characteristics. See our OSF page for more detail https://osf.io/6s43f/?view_only=a152fed51f8f4030b8690422dd8db517.
